# Elevated average maximum intrabolus pressure on high-resolution manometry is associated with esophageal dysmotility and delayed esophageal emptying on timed barium esophagram

**DOI:** 10.1186/s12876-022-02165-5

**Published:** 2022-02-21

**Authors:** Katelyn E. Madigan, J. Shawn Smith, Joni K. Evans, Steven B. Clayton

**Affiliations:** 1 Department of Medicine, Wake Forest School of Medicine, Medical Center Boulevard, Winston-Salem, NC 27157 USA; 2 Department of Medicine, Section on Gastroenterology, Wake Forest School of Medicine, Winston-Salem, USA; 3 Department of Medicine, Prisma Health, Greenville School of Medicine, Greenville, USA; 4 Department of Public Health Sciences, Wake Forest School of Medicine, Winston-Salem, USA; 5 Department of Medicine, Section on Gastroenterology, Greenville School of Medicine, Greenville, USA

**Keywords:** Esophageal dysmotility, High-resolution manometry, Timed barium esophagram

## Abstract

**Background:**

Intrabolus pressure (IBP) recorded by high-resolution manometry (HRM) portrays the compartmentalized force on a bolus during esophageal peristalsis. HRM may be a reliable screening tool for esophageal dysmotility in patients with elevated average maximum IBP (AM-IBP). Timed barium esophagram (TBE) is a validated measure of esophageal emptying disorders, such as esophagogastric junction outflow obstruction and achalasia. This study aimed to determine if an elevated AM-IBP correlates with esophageal dysmotility on HRM and/or delayed esophageal emptying on TBE.

**Methods:**

A retrospective analysis of all HRM (unweighted sample n = 155) performed at a tertiary referral center from 09/2015–03/2017 yielded a case group (n = 114) with abnormal AM-IBP and a control group (n = 41) with a normal AM-IBP (pressure < 17 mmHg) as consistent with Chicago Classification 3. All patients received a standardized TBE, with abnormalities classified as greater than 1 cm of retained residual liquid barium in the esophagus at 1 and 5 min or as tablet retention after 5 min.

**Results:**

AM-IBP was significantly related to liquid barium retention (p = 0.003) and tablet arrest on timed barium esophagram (p = 0.011). A logistic regression model correctly predicted tablet arrest in 63% of cases. Tablet arrest on AM-IBP correlated with an optimal prediction point at 20.1 mmHg on HRM. Patients with elevated AM-IBP were more likely to have underlying esophageal dysmotility (95.6% vs. 70.7% respectively; p < 0.001), particularly esophagogastric junction outflow obstruction disorders. Elevated AM-IBP was associated with incomplete liquid bolus transit on impedance analysis (p = 0.002).

**Conclusions:**

Our findings demonstrate that an elevated AM-IBP is associated with abnormal TBE findings of esophageal tablet retention and/or bolus stasis. An abnormal AM-IBP (greater than 20.1 mm Hg) was associated with a higher probability of retaining liquid bolus or barium tablet arrest on TBE and esophageal dysmotility on HRM. This finding supports the recent incorporation of IBP in Chicago Classification v4.0.

## Introduction

Esophageal bolus transit is a coordinated physiologic process in which esophageal smooth muscle contractility, intrabolus pressure (IBP), and deglutitive lower esophageal sphincter (LES) relaxation {integrated relaxation pressure (IRP)} determine the effectiveness of swallowing [1–3].

**Fig. 1:**
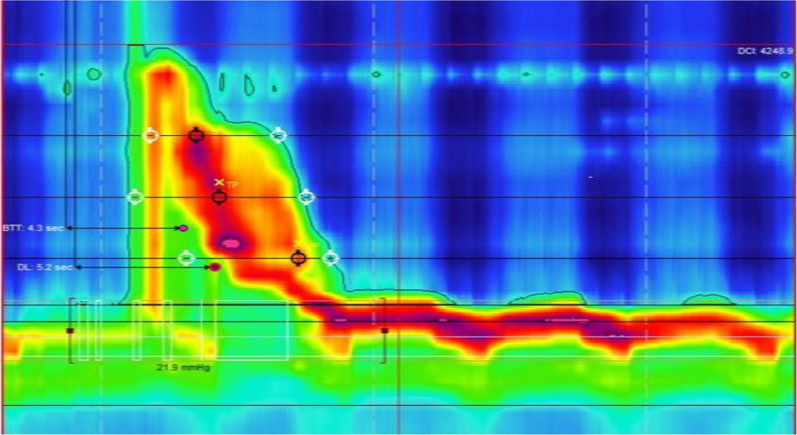
Patient with esophagogastric outflow obstruction on high-resolution manometry with intrabolus pressure > 27 mmHg

IBP is the measurement of the compartmentalized pressure exerted on a solid or liquid bolus transiting through the esophagus, as shown in Fig. 1 [1–3]. Elevated IBP can be an indirect indicator of esophageal obstruction. However, it is unknown if average maximum IBP (AM-IBP) correlates with esophageal dysmotility on HRM and delayed esophageal emptying on TBE, particularly in the advent of Chicago Classification version 4 (CCv4) in 2021 [3, 4]. The previous conventional manometric equipment lacked the precision to measure dynamic IBP variations accurately [5, 6]. High-resolution manometry (HRM) provides a reliable screening tool for the initial detection of esophageal dysmotility [4, 7]. An increased number of recording sites with HRM creates a spatial continuum of intraluminal pressure through the interpolation between adjacent sensors [8, 9]. Timed barium esophagram (TBE) is a validated assessment to be used in conjunction with HRM for quantifying esophageal emptying in disorders causing esophageal outflow obstruction, such as EGJOO and achalasia [10–12].

The calculation and clinical utility of AM-IBP was first described by Niebisch et al. in 2013. The AM-IBP is calculated 1 cm above the proximal border of the EGJ and displayed as the maximal average pressure of 3 nonconsecutive seconds [3–16]. AM-IBP can be a surrogate marker for an obstructive process at the esophagogastric junction (EGJ). We hypothesized that AM-IBP would correspond to esophageal dysmotility on HRM and poor esophageal emptying on TBE. The objective of this study was to help fill an important gap in the literature by evaluating the relationship between elevated AM-IBP, esophageal dysmotility on HRM, and poor esophageal emptying on TBE.

## Materials and methods

### Study design and data source

This was a retrospective cross-sectional analysis of all patients undergoing HRM for non-obstructive dysphagia symptoms performed at a tertiary referral center from September 2015 to March 2017.

### Study subjects

Inclusion criteria included adult patients (ages 18 and over) for an unweighted sample size of n = 155 patients. We compared two groups: a case group of patients with an abnormal AM-IBP reading (n = 114 patients) and a control group with a normal AM-IBP reading (n = 41 patients). Niebisch et al. described 14.6 mmHg as the upper 95% value for healthy volunteers; therefore, we defined abnormal AM-IBP as IBP > 17 mmHg in accordance with the ManoView ™software (Sierra Scientific Instruments Inc., Version 3.0, Los Angeles, CA, USA) [16]. All patients in both these groups also underwent a TBE study. In the initial unweighted sample of 157 patients, there were two patient exclusions to note due to intolerance: one patient did not receive measurements of the barium column, and another patient did not have measurements recorded at one minute. Further details of the specific study protocol are outlined below.

### High-resolution manometry (HRM) protocol

We studied the esophageal motor function of all patients with the same 36-channel solid-state catheter system (Medtronic, Inc., Minneapolis, MN, USA) with high fidelity circumferential sensors at 1 cm intervals, as well as intra-luminal impedance. The manometry catheter with five intragastric sensors was placed trans-nasally and positioned to record from the hypopharynx to the stomach. After confirming patients had performed at least 6 h of fasting before the test, patients were placed in the supine position. The protocol included a 3-min baseline period and at least ten 5-mL water swallows, each separated by 30-s intervals. Pertinent metric data recorded includes lower esophageal sphincter basal pressure, intrabolus pressure (IBP), integrated relaxation pressure (IRP), distal contractile latency (DCL), large breaks, frontal contractile velocity, and distal latency (DL). All data were analyzed using ManoView version 3.0 software in the high-resolution esophageal color topography mode for standardized data analysis. Studies were interpreted according to Chicago Classification version 3.0. In addition, to the IRP, basal LES sphincter pressure was recorded and considered abnormal if outside the accepted normative values (13–-43 mmHg). Earlier versions of ManoView did routinely report AM-IBP. The AM-IBP was expressed as the average pressure > 2 mmHg during deglutitive relaxation. AM-IBP is also measured relative to gastric pressure and calculated as the mean of the maximum pressure of three non-continuous seconds. Additionally, water-perfused HRM systems are available but were not used in this study [17].

### Timed barium esophagram (TBE) protocol

TBE is a standardized protocol with the administration of 240 mL (8 oz) of low-density barium while the patient is standing upright. We obtained spot films at 1 min and 5 min to assess liquid emptying from the esophagus. Barium column height was measured from the gastroesophageal (GE) junction to the height of the top of the column, with abnormal tests being the retention of liquid at 1 min and five minutes. Following this measurement, the patient cleared their esophagus with water before ingesting a 13-mm barium tablet. The barium height and width in centimeters were recorded at 1 min and 5 min, with an abnormal test defined as the retention of the tablet at 5 min.

### Statistical analysis

Continuous data were expressed as means with standard deviations, and we made comparisons with the Student’s T-test with initial statistical analysis performed using Microsoft Excel (version 1809. Discrete data were recorded as proportions, and we made comparisons using Chi-square tests. Two-sided p-values of 0.05 or less were considered significant. Additional statistical analysis was performed with logistic regression to evaluate the associations between AM-IBP and tablet arrest or barium height.

The following variables were included in a multivariable logistic regression model to help predict variables associated with abnormal TBE: BMI, Mean LES RBP, mean LES RP, LES IBP AM-IBP, Hypertensive LES, Type I-III Achalasia, EGJOO, combined other dysmotility (jackhammer esophagus, ineffective esophageal motility, diffuse esophageal spasm, hypotensive LES and pseudoachalasia), hiatal hernia and TBE tablet arrest.

## Results

### Baseline characteristics

**Fig. 2:**
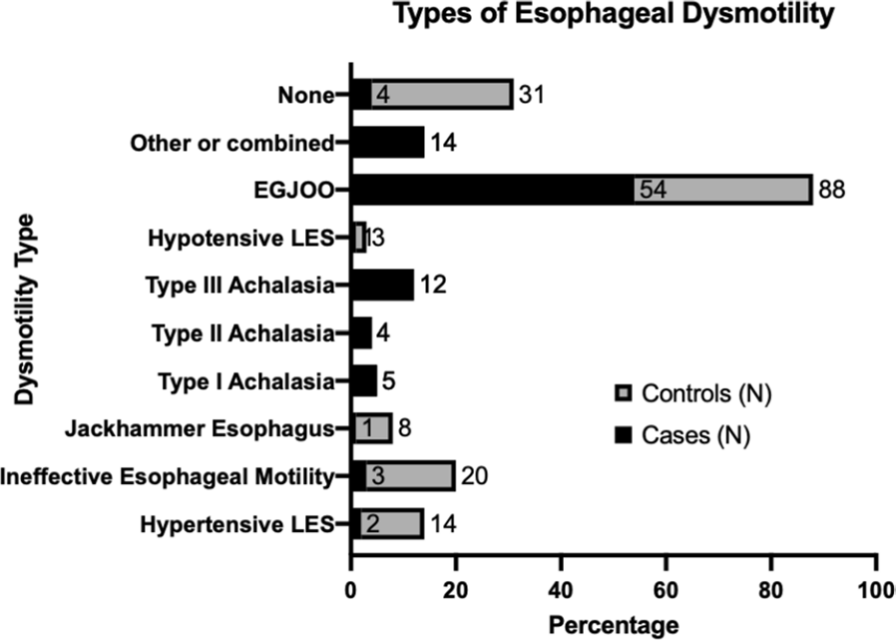
Distribution of esophageal motility disorder by subtype, as consistent with Chicago classification 3.0 at time of data collection. In addition to disorders described according to CCv3.0, basal LES sphincter pressure was recorded and considered abnormal if it was outside the accepted normative values (13–43 mmHg)

Of the 155 patients tested between September 2015 and March 2017, the vast majority (114 patients) were found to have abnormal AM-IBP on HRM. There were 41 patients with normal AM-IBP in the control group, as shown in Table 1. The population’s average age was 58 years ± 13.2 years with an average BMI of 30.4 ± 6.87; there were no significant differences in age or BMI between the groups. Significantly more females were in the control group compared to the case group (80.5% vs. 58.8%; p = 0.01). As outlined in Table 2, there was significantly more abnormal manometry testing in the case group than in the control group (95.6% vs. 70.7% respectively; p < 0.001). Figure 2 outlines the distribution of esophageal motility disorder by subtype, as per classification at the time of data collection. In addition to conditions described according to CCv3.0, basal LES sphincter pressure was recorded and considered abnormal if it was outside the accepted normative values (13–43 mmHg).

**Table 1:** Manometry and timed barium esophagram (TBE) in case-cohort and control-cohort

Manometry	Cases	Controls	p-value
Number of patients	114	41	
Age (years); Mean ± SD	61.3 ± 13.0	55.1 ± 15.4	0.75
BMI; Mean ± SD	29.9 ± 5.5	28.5 ± 6.7	0.75
Sex, N (%)			0.01
Male	47 (41.2%)	8 (19.5%)	
Female	67 (58.8%)	33 (80.5%)	
Hernia, N (%)	34 (29.8%)	20 (48.8%)	0.003
Size (cm); Mean ± SD	2.9 ± 1.9	3.0 ± 1.4	0.89

TBE	Cases	Controls	p-value
Total, N	114	41	
Normal	40	29	< 0.001
Abnormal^a^	74	12	
Tablet arrest, N (%)	41 (36.0%)	4 (9.8%)	< 0.001
Hiatal hernia, N (%)	5 (4.4%)	4 (9.8%)	< 0.001
Stricture, N (%)	3 (2.6%)	0 (0%)	–
TBE 1' N			
Column > 0 mm, N (%)	54 (48.2%)	10 (24.4%)	0.003
Mean ± SD (mm)	53 (73.9%)	27 (59.4%)	0.43925
TBE 5' N			
Column > 0 mm, N (%)	37 (32.7%)	2 (4.9%)	< 0.001
Mean ± SD (mm)	26.3 (49.3)	2.4 (13.1%)	0.44

^a^Please see Fig. 2 for further elaboration of the classification of abnormal subtypes

**Table 2:** Manometry data

Manometry data	Cases	Controls	p-value
Number (N)	114	41	
Mean LES RBP (mmHg)**			0.19
Normal, N (%)	53 (46.5%)	24 (58.5%)	
Abnormal, N (%)	61 (53.5%)	17 (41.5%)	
Mean ± SD	45.7 (21.2%)	34.5 ± 17.1	0.48
Mean LES RP (mmHg)^			< 0.001
Normal, N (%)	23 (20.2%)	23 (56.1%)	
Abnormal, N (%)	91 (79.8%)	18 (43.9%)	
Mean ± SD	22.3 ± 11.0	13.3 ± 7.5	0.48
DCI (mmHg-cm-s)^o^			0.05
Normal, N (%)	76 (66.7%)	34 (82.9%)	
Abnormal, N (%)	38 (33.3%)	7 (17.1%)	
Mean ± SD	3708.8 ± 3952.9	2116.5 ± 2079.3	0.33
LES IBP (mmHg)^x^			< 0.001
Normal, N (%)	55 (48.2%)	35 (85.4%)	
Abnormal, N (%)	59 (51.8%)	6 (14.6%)	
Mean ± SD	7.4 ± 7.1	3.0 ± 4.6	0.44
LES AVG MAX (mmHg)^z^			0.95
Normal, N (%)	N/A	41	
Abnormal, N (%)	114	N/A	
Mean ± SD	22.6 ± 12.4	12.7 ± 3.1	
Incomplete bolus clearance			0.002
Normal, N (%)	49 (43.4%)	29 (70.7%)	
Abnormal, N (%)	64 (56.6%)	12 (29.3%)	
Manometry			< 0.001
Normal, N (%)	5 (4.4%)	12 (29.3%)	
Abnormal, N (%)	109 (95.6%)	29 (70.7%)	

Normal HRM on TBE was defined as column height < 17 mmHg, as measured from gastroesophageal junction to the height of the top of the column

*BMI: body mass index

**Mean lower esophageal sphincter respiratory basal pressure

^Mean lower esophageal sphincter residual pressure

^o^Distal contractile integral

^x^Lower esophageal sphincter intrabolus pressure

^z^Average maximum lower esophageal sphincter pressure in mmHg

### Timed barium esophagram

**Fig. 3:**
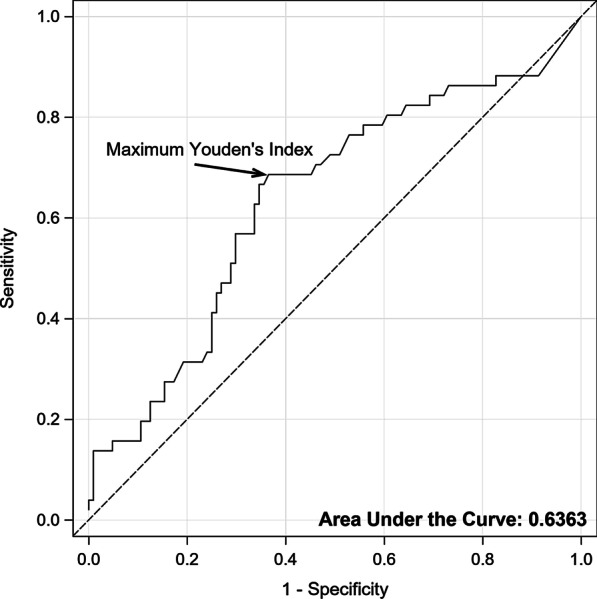
Receiver operating curve for optimal prediction point for tablet arrest at an average max IBP at 20.1 mm Hg. Additionally, the AUC for AM-IBP, as shown correctly predicted tablet arrest in 64% of patients

Case-patients were found to have significantly more abnormalities reported on TBE than control-patients (64.9% vs. 29.1% respectively; p < 0.001). As outlined in Table 1, a significant difference remained between the two groups in the presence of hiatal hernias (p < 0.001). There was a higher frequency of patients with retention of the barium tablet in the case group compared to the control group (p < 0.001). A higher proportion of case-patients than control-patients had retention of liquid barium at one minute (48.2% vs. 24.4%; p = 0.003). More case-patients exhibited barium retention at five minutes compared to control-patients (32.7% vs. 4.9%; p < 0.001). Additionally, AM-IBP was significantly related to tablet arrest (p = 0.011) in a logistic regression model, which correctly predicted tablet arrest in 64% of patients. An optimal prediction point, found by maximizing Youden’s index, was associated with an average max IBP of 20.1 mm H (Fig. 3). Of those with tablet arrest, 69% had an average maximum IBP ≥ 20.1 mm Hg. We noted no differences of significance in the quantitative measurements of the column heights amongst the two groups.

A backward elimination procedure evaluating EGJOO subsets resulted in a model revealing abnormal TBE is more often associated with both tablet arrest and Types I-III achalasia. Specifically, patients with abnormal TBE were found to have a 62% higher likelihood for TBE tablet retention compared to those with normal TBE (p < 0.001).

### High-resolution impedance manometry

A higher percentage of case-patients vs. control-patients had abnormal manometries (95.6% vs. 70.7% respectively; p < 0.001). The proportion of abnormal manometric findings were significantly different between the two groups in mean lower esophageal sphincter respiratory basal pressure, mean average lower esophageal sphincter residual pressure, distal contractile integral, intrabolus pressure at the lower esophageal sphincter, and incomplete bolus clearance (Table 2). We also found significance with a higher frequency of tablet retention in the case group than in the control group (88.8% vs. 64.9% respectively; p = 0.015). A hiatal hernia was present in more people in the control group than in the case group (48.8% vs. 29.8% respectively; p = 0.003).

Liquid bolus transit measured by impedance was more likely to be complete vs. incomplete in patients with a normal AM-IBP versus patients with elevated AM-IBP (p = 0.002).

## Discussion

Our significant findings were AM-IBP correlated with tablet arrest and liquid bolus retention on timed barium esophagram, which maintained significance by logistic regression. Similarly, to liquid barium retention on TBE, elevated AM-IBP was associated with incomplete liquid bolus transit during impedance analysis with HRM.

Specifically, an optimal prediction point for AM-IBP was found at 20.1 mmHg. Additionally, we found that both anatomical and clinically relevant EGJOO patients exhibit elevated IBP manometrically. EGJOO is a heterogeneous collection of esophageal dysmotility types with manometry characterization crucial in the diagnosis. In the updated CCv.4, clinical correlation merits higher consideration to allow for improved prognostication of clinical outcomes. In what we have found to be the largest cohort of patients with elevated IBP, this association can help diagnose clinically relevant esophageal obstructive disorders, particularly in the context of the updated CCv4.0 metrics recognizing the utility of IBP.

As a marker of flow impedance within the esophagus, AM-IBP proves to be a novel and objective metric in grading the severity of other conditions known to cause strictures or similar changes to the functionality of the esophagus [17]. IBP can be an indirect measure of the distensibility of the distal esophagus or the EGJ [18–21]. The AM-IBP measurement recorded by HRM demonstrates the compartmentalized force exerted on a bolus during complete esophageal peristalsis and is complementary to IRP in characterizing disorders of EGJOO [3, 16, 18, 22] Our study is consistent with findings previously reported for elevated IBP in EGJOO [23–27]. For example, a survey by Scherer et al*.* examined all the composite characteristics of patients with post-Nissen fundoplication (n = 8) and patients with functional obstruction (n = 16) and found a higher frequency of maximum IBP (defined as IBP > 30 mmHg) in patients with post-Nissen fundoplication when compared to patients with functional obstruction (n = 63 swallows; 79% vs. n = 86 swallows; 54%, respectively) [28].

Continued refinement in diagnostics has led to improved metrics to allow for a reliable and reproducible evaluation of esophageal motility. With the advent of HRM, additional information about obstructive forces near the EGJ beyond conventional manometry is available due to the number and spacing of solid-state pressure sensors in HRM [9, 12, 22, 26]. This increased number of sensors in HRM essentially eliminates a deficiency of conventional manometric systems: the problem of movement-related artifacts [8, 27, 29].

CCv4.0 provides a more robust characterization of outflow disorders such as EGJOO in a more patient/symptom-oriented protocol [5, 6]. Maneuvers in various positions in combination with clinical symptoms offer a more specific depiction. Therein, incorporating IBP in diagnosing disorders of EGJOO allows for a more precise diagnosis to mitigate over-diagnosis secondary to artifacts.

There are limitations to our study to be acknowledged. First, we analyzed retrospective data at a single center; thus, results may not reflect other centers. However, consistent with our hypothesis and literature to date, higher intrabolus pressures are commonly found with disorders of outflow obstruction (more minor with different subtypes) [30–33]. Second, since this data gathering, modalities such as endoluminal functional lumen imaging probe (EndoFLIP™ Medtronic Minneapolis, MN 55432) have become more prevalent in diagnosing EGJ diseases; EndoFLIP™ was not performed during this study [34, 35]. The majority of patients referred for HRM had clinical symptoms concerning for non-obstructive dysphagia, which is an inherent selection bias. The data analyzed included a sample size that can be expanded upon in future studies. Finally, despite continual improvements, a limitation to acknowledge is the lack of safeguards in the ManoView v.3.0 software metric's susceptibility to erroneous pressure artifact. Nonetheless, our study provides results for evaluating the association between AM-IBP barium retention on timed barium esophagram and how it relates to disorders of outflow obstruction. Our study validates the clinical utility of IBP as an important manometric measure and supports its incorporation in CCv4.0.

## Conclusion

AM-IBP in the interpretation of HRM can be a surrogate marker for resistance and obstruction in the esophagus, particularly in EGJOO subsets [36–39]. Measuring esophageal stasis is vital for disorders of esophageal gastrojunctional outflow obstruction [40-42]. An abnormal AM-IBP with an optimal prediction point of 20.1 mm Hg was associated with a higher probability of retaining liquid bolus or barium tablet arrest on TBE and esophageal dysmotility on HRM. Future studies can further characterize how elevated intrabolus pressure correlates to poor bolus transit in the classification of esophageal dysmotility.

## Data Availability

The datasets generated and/or analyzed during the current study are not publicly available due to hospital patient confidentiality restrictions due to potentially identifying information, but are available from the corresponding author on reasonable request.
